# Prognostic value of inflammatory markers NLR, PLR, LMR, dNLR, ANC in melanoma patients treated with immune checkpoint inhibitors: a meta-analysis and systematic review

**DOI:** 10.3389/fimmu.2024.1482746

**Published:** 2024-10-18

**Authors:** Yan Ou, Shufang Liang, Qiangqiang Gao, Yongran Shang, Junfang Liang, Weitao Zhang, Sha Liu

**Affiliations:** ^1^ Department of Plastic and Aesthetic Surgery, Affiliated Hospital of Shaanxi University of Chinese Medicine, Shaanxi, China; ^2^ Department of Burns and Plastic Surgery, 969th Hospital of PLA Joint Logistic Support Force, Inner Mongolia, China; ^3^ Department of Proctology, Affiliated Hospital of Shaanxi University of Chinese Medicine, Shaanxi, China; ^4^ The First Clinical Medical College of Shaanxi University of Chinese Medicine, Shaanxi, China

**Keywords:** melanoma, immune checkpoint inhibitors, inflammatory markers, survival, meta-analysis

## Abstract

**Background:**

Immune checkpoint inhibitors (ICIs) are an emerging tumor treatment pathway after traditional surgery, chemoradiotherapy, and targeted therapy. They have proven to be effective in a variety of cancers, but may not respond to non-target populations. Inflammatory markers such as neutrophil to lymphocyte ratio (NLR), platelet to lymphocyte ratio (PLR), lymphocyte to monocyte ratio (LMR), derived neutrophil lymphocyte ratio (dNLR), and neutrophil count (ANC) have been shown to be strongly associated with tumor prognosis, but their prognostic significance remains controversial. We therefore performed a meta-analysis to explore the association between NLR, PLR, LMR, dNLR, ANC and prognostic and clinicopathological factors in melanoma patients treated with ICIs.

**Methods:**

A comprehensive search was conducted in Pubmed, Embase, Web Of Science and Cochrane databases, and the last search time was July 2024. To estimate the prognostic value of NLR, PLR, LMR, dNLR, ANC for PFS and OS, hazard ratio (HR) and corresponding 95% confidence interval (CI) estimates were used.

**Results:**

This meta-analysis ultimately included 22 cohort studies involving 3235 melanoma patients. Meta-analysis results showed that high levels of NLR in melanoma patients receiving ICIs were associated with poorer OS and PFS, Merging the HR respectively OS [HR = 2.21, 95% CI (1.62, 3.02), P < 0.001], PFS [HR = 1.80, 95% CI (1.40, 2.30), P < 0.001]; High levels of PLR were associated with poor OS and PFS, and the combined HR was OS[HR=2.15,95%CI(1.66,2.80),P < 0.001] and PFS[HR=1.67,95%CI(1.31,2.12),P < 0.001]. High levels of dNLR were associated with poor OS and PFS, with combined HR being OS[HR=2.34,95%CI(1.96,2.79),P < 0.001] and PFS[HR=2.05,95%CI(1.73,2.42),P < 0.001], respectively. High ANC was associated with poor OS and PFS, and combined HR was OS[HR=1.95,95%CI(1.16,3.27),P < 0.001] and PFS[HR=1.63,95%CI(1.04,2.54),P=0.032], respectively. Increased LMR was associated with prolonged OS and PFS, with combined HR being OS[HR=0.36, 95%CI(0.19,0.70),P < 0.001] and PFS[HR=0.56,95%CI(0.40,0.79),P=0.034], respectively.

**Conclusion:**

In melanoma patients treated with ICIs, elevated levels of NLR, PLR, dNLR, and ANC were associated with poorer overall survival OS and PFS. Conversely, a high LMR correlated with improved OS and PFS. Subgroup analyses indicated that dNLR may be linked to a worse prognosis in melanoma patients. In summary, inflammatory markers such as NLR, PLR, LMR, dNLR, and ANC serve as effective biomarkers for the prognostic assessment of melanoma patients following ICI treatment. These markers provide valuable insights for treatment decision-making in the realm of melanoma immunotherapy, and we anticipate further high-quality prospective studies to validate our findings in the future.

**Systematic review registration:**

https://www.crd.york.ac.uk/PROSPERO/#recordDetails, identifier CRD42024573406.

## Introduction

Skin cancer is the most common type of cancer worldwide, with over 1.5 million new cases reported in 2020. Melanoma, which arises from the malignant transformation of melanocytes in the skin, mucous membranes, and other tissues, is the most prevalent form of skin cancer, accounting for approximately one-fifth of all skin cancer cases. In 2020, there were 325,000 new melanoma cases and 57,000 melanoma-related deaths globally. The incidence and mortality rates are higher in men than in women. By 2040, the number of new melanoma cases is projected to increase to 510,000, representing a 50% increase, while melanoma-related deaths are expected to rise to 96,000, indicating a 68% increase (1).Due to its high malignancy and tendency to metastasize through lymphatic and hematogenous routes, the overall efficacy of conventional surgical and radiotherapeutic interventions is limited. As a result, the mortality rate for patients with advanced melanoma exceeds 75%, and the 5-year survival rate is less than 15% (2, 3).Immunotherapy signifies a departure from traditional chemotherapy and targeted therapy by emphasizing the activation of the anti-tumor response of immune cells, rather than directly targeting and destroying cancer cells. Its primary objective is to bolster the immune system’s capacity to eradicate malignant cells. Immune checkpoint blockade (ICB) is an immunotherapeutic strategy that utilizes immune checkpoint inhibitors (ICIs) to prevent immune checkpoints, such as cytotoxic T-lymphocyte antigen 4 (CTLA-4), programmed death 1 (PD-1), lymphocyte activation gene 3 (LAG-3), and T-cell immunoglobulin and ITIM domain (TIGIT), from binding to their respective ligands. This approach seeks to reinvigorate suppressed immune cells and enhance their capacity to eliminate tumors, thereby restoring their anti-tumor efficacy (4). In recent years, immune checkpoint inhibitors (ICIs) have demonstrated remarkable efficacy in tumor treatment by enhancing the signaling cascade of T-cell function, thereby promoting immune activation and causing damage to tumor tissues, and several studies have confirmed that ICIs have shown good anticancer activity in a variety of cancers such as melanoma (5), gastric cancer (6), non-small-cell lung cancer (7), renal cell carcinoma (8), esophageal cancer (9), and hepatocellular carcinoma (10). ICIs have demonstrated considerable promise in the treatment of malignant melanoma, significantly enhancing both overall survival (OS) and progression-free survival (PFS) among patients with advanced melanoma. A cohort study (11) involving 16,831 patients revealed that the overall survival of stage IV melanoma patients undergoing first-line ICI treatment was markedly improved. Specifically, the overall survival for patients treated with ICIs was 43.7 months, in contrast to 16.1 months for those receiving chemotherapy or targeted therapy, with a 2% year-on-year decline in mortality rates for melanoma patients between 2016 and 2020 (12). Currently, the U.S. FDA has approved three ICIs that target distinct molecules: PD-1, PD-L1, and CTLA-4. The approved drugs include Ipilimumab, Pembrolizumab, Nivolumab, Cemiplimab, Atezolizumab, and Durvalumab.

While immune checkpoint inhibitors, particularly PD-1 antibodies, have demonstrated promising anti-tumor effects in the clinical treatment of melanoma, some patients do not respond to this therapy. A study of a clinical trial of PD-1 antibodies in advanced solid tumors by Suzanne L Topalian (13) demonstrated that the cumulative response rate in patients with non-small-cell lung cancer was 18%, melanoma 28%, and renal cell carcinoma 27%. This indicates that a significant number of tumor patients do not benefit from immune checkpoint blockade therapy. In addition, ICIs, while activating T cells to attack the tumor, may also trigger irAEs, affecting patients’ quality of life and treatment compliance (14). Therefore, identifying biomarkers to predict the efficacy of immune checkpoint inhibitors (ICIs) and accurately screening patients who will benefit from them represents an urgent challenge in the field of immunotherapy. Current established immunotherapy biomarkers include PD-L1 expression, tumor mutation burden (TMB), and microsatellite instability (MSI). However, these biomarkers encounter several issues, including high testing costs, difficulties in sampling advanced patients, and the absence of uniform and clear cut-off values, which limit their clinical utility (15). Immunosuppression in the tumor microenvironment is a known factor that promotes tumor growth and cancer cell migration, induced by systemic and chronic inflammation, and mediated by several circulating cells (16),numerous studies have demonstrated that chronic inflammation, mediated by inflammatory cytokines, significantly influences tumor development. Tumorigenesis, progression, metastasis, and prognosis are closely linked to the body’s inflammatory state and immune function. Systemic inflammation facilitates tumor growth and metastasis through the production of pro-inflammatory molecules by innate immune cells, as well as the activation of oncogenic signaling pathways (17). A substantial body of research has established the predictive significance of inflammatory markers such as NLR (18), PLR (19), and LMR (20) in melanoma. A study conducted by Schneider et al. (21) demonstrated that an NLR of ≥4, a PLR of ≥145, and an LMR of <2 were significantly associated with a reduced incidence of overall survival (OS). These markers of preoperative peripheral inflammation function as indicators of poor prognosis in melanoma patients undergoing surgical intervention. Furthermore, a meta-analysis by Zhan et al. (22) corroborated these findings, indicating that elevated preoperative NLR is linked to poor prognosis in melanoma patients, suggesting that NLR may play a critical role in the prognostic assessment of this patient population. Several studies have demonstrated that peripheral blood markers, including NLR, dNLR, PLR, LMR, and ANC, can reflect systemic inflammation, and that they are noninvasive, economical, simple, inexpensive, and easily available, and have been used to reflect the immune and inflammatory status of patients with various malignant tumors (23–25), This is advantageous for clinical diagnosis and prognostic assessment of cancer. Currently, the role of inflammatory markers in predicting the survival response of melanoma to ICIs remains contentious, and there is a lack of systematic meta-analyses focusing on inflammatory markers such as NLR, dNLR, PLR, LMR, and ANC. Therefore, we conducted this meta-analysis to evaluate the prognostic significance of these inflammatory markers in melanoma patients undergoing ICI treatment. The objective is to enhance the ability of clinicians to accurately predict the response of melanoma patients to ICIs, thereby facilitating personalized treatment options and establishing a foundation for future research.

## Materials and methods

The protocol has been registered in the International Prospective Register of Systematic Reviews data base (PROSPERO: CRD42024573406).

### Literature search strategy

Two researchers (OY NAD LSF) independently conducted the search using Pubmed, Embase, Web Of Science, and Cochrane databases. Mesh words in PubMed are used to expand the search scope and include: Melanoma, Malignant Melanoma, Melanomas, Neutrophil-to-lymphocyte ratio, Platelet-lymphocyte ratio, “lymphocyte-monocyte ratio”, “Derived neutrophil to lymphocyte ratio”, “Absolute neutrophil count”, “Immune Check point Inhibitor”, “Immune Check point Blockers”, “PD-L1Inhibitors”, “Programmed Death-Ligand1Inhibitors”, “Ipilimumab”, “Pembrolizumab”, “Tremelimumab”, “Nivolumab”. There are no restrictions on language and type of research in the search strategy, and the last time to search is July 1, 2024. The two researchers screened the papers based on title, abstract and inclusion criteria. Two researchers respectively extracted and reviewed the basic information of relevant literature, research objectives, results and follow-up data. If there was any disagreement, third-party experts would evaluate it. The systematic review was conducted in accordance with the Preferred Reporting Project for Systematic Review and Meta-Analysis (PRISMA) guidelines.

### Inclusion and exclusion criteria

The inclusion criteria are as follows:

(1) Clinically diagnosed melanoma patients who have received ICIs treatment;

(2) To report the effects of high and low expression of inflammatory markers NLR, PLR, LMR, dNLR, ANC on patient outcomes, using hazard ratio (HR) and 95% confidence interval (CI) studies;

(3)They were divided into exposed group (high expression of inflammatory markers) and non-exposed group (low expression of inflammatory markers) according to their exposure.

(4) Chinese and English literature;

(5) Outcome measures were overall survival (OS) or progression-free survival (PFS);

(6) The included study design was randomized controlled trial, observational study, cross-sectional study, retrospective study or prospective study.

The exclusion criteria are as follows:

(1) The type of disease research or intervention is not consistent;

(2) No prognostic survival information was provided;

(3) Outcome indicators cannot be extracted;

(4) repeated publication or incomplete information;

(5) non-comparative studies, animal experiments, reviews, letters, guidelines, case reports, pathological mechanisms, conference abstracts, expert opinions, editorials, reviews;

(6) Documents in other languages.

### Data extraction

Two researchers independently screened the literature according to the inclusion and exclusion criteria, information was independently extracted using a standardized data extraction form, cross-checked individually by both researchers, and disagreements were resolved through discussion. Studies were excluded if relevant data were not available. For each study, the following information was collected: (1) study characteristics: first author, country, year of publication, type of study, study duration, tumor clinical stage, type of survival analysis, immune checkpoint inhibitors used, and critical values; (2) patient baseline: number of patients, age, and gender; and (3) Research results: If the HR values of OS and PFS are described in this paper, they are extracted directly; if OS and PFS are described in Kaplan-Meier graphs, Engauge Digitizer is used for conversion extraction.

### Literature quality assessment

The quality of the included cohort studies was independently assessed using the Newcastle-OttawaScale (NOS), which consists of three metrics: cohort selection, comparability, and outcome assessment. The modified NOS is a 9-point scale, with low-quality studies scoring 1-3, moderate-quality 4-6, and high-quality 7-9. Scoring was done independently by two investigators, and third-party experts were consulted to resolve any large differences between their scores or if this affected the study’s inclusion in the final analysis.

### Statistical analysis

StataSE15.0 software was used for statistical analysis to calculate the combined HR and 95% confidence interval (95% CI), and P<0.05 showed a significant difference between the two groups. Heterogeneity was evaluated using I² values,I²≤30%,30%<I²<75% and ≥75% were considered to indicate low, medium and high heterogeneity, respectively.I²<50% was analyzed using a fixed-effects model, while I²≥50% was analyzed using a random-effects model. Sensitivity analysis was carried out on the results with large heterogeneity, one study in the merger was excluded one by one, the combined effect size and heterogeneity changes of the remaining literature were evaluated, and the source of heterogeneity was analyzed. Begg’s funnel plot and Egger’s test were used to evaluate whether there was publication bias, and no publication bias existed if P > 0.05.

## Results

### Literature search results

**Figure 1:**
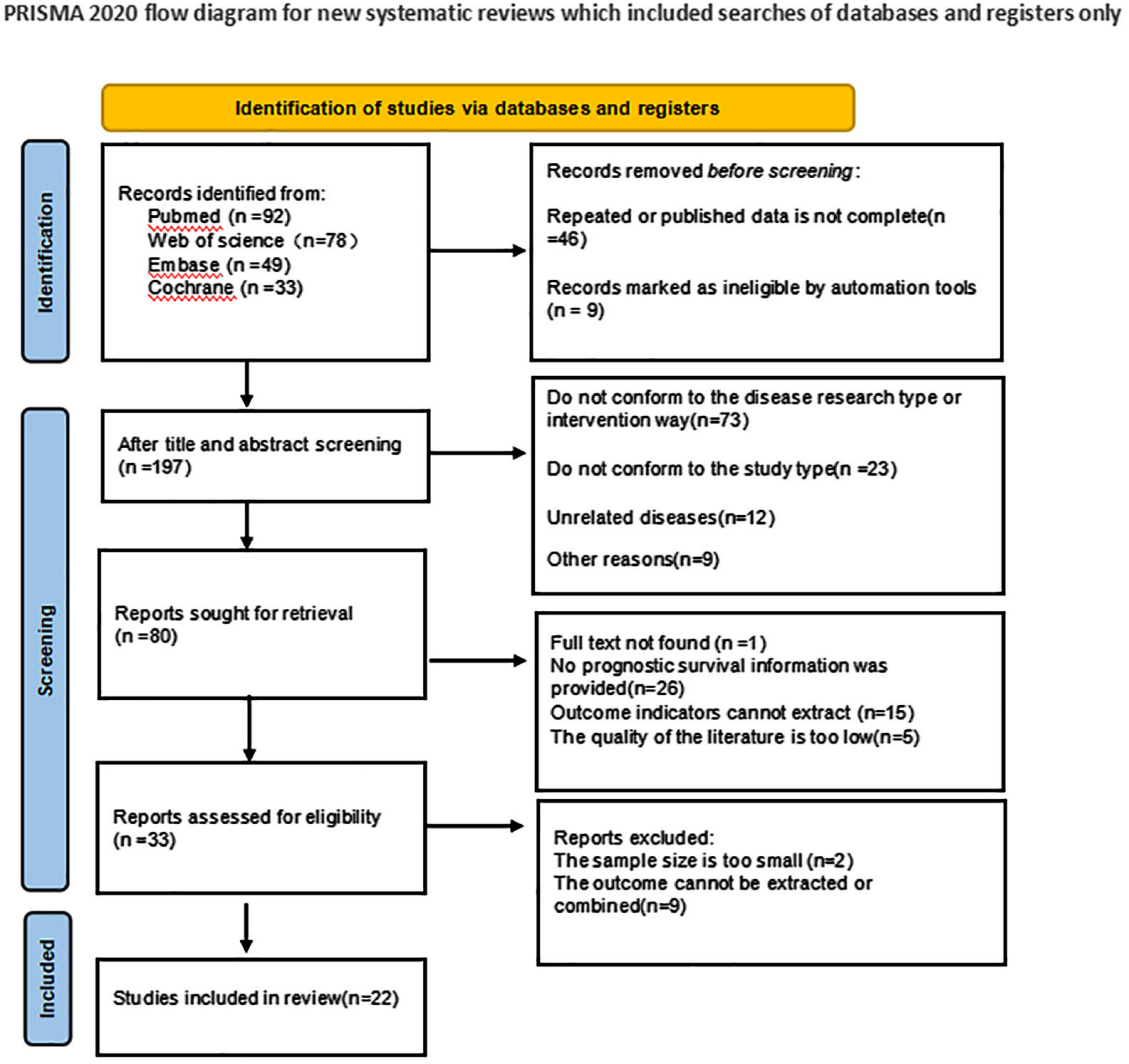
Schematic diagram of literature search criteria and including studies in meta-analyses.

In the initial literature search, a total of 252 articles were searched. 46 duplicate studies were excluded; After reading the title and abstract of the article, 172 studies were excluded according to the exclusion criteria, and 80 studies were initially included. We then read the full text and excluded 58 studies that did not meet the inclusion criteria. Finally, 22 studies were included in the meta-analysis. The literature screening process and results are shown in **Figure 1**.

### Basic characteristics of the included studies

As shown in **Table 1**, the 22 studies included evaluated a total of 3235 patients with melanoma after ICIs treatment. All 22 studies were cohort studies, of which 18 were retrospective cohort studies and 4 were prospective cohort studies. Multiple inflammatory markers were studied in one study in the included literature, so we numbered different inflammatory markers in the same literature. Study characteristics, patient baseline, and study results of the included studies are shown in **Table 1**.

**Table 1:** Clinical and demographic characteristics of studies included in the meta-analysis.

First author	Year	Research type	Author states	Sample size	Age	Sex(Male/female)	Duration	Clinical stage	ICIs agents	Follow-up (months)	Survival analysis	Cut-off	Inflammatory factor type	Survival outcome
Muhammad Z1 (26)	2019	Cohort (retrospective)	Lebanon	120	64 (23–92)	76/44	2011-2017	IIIC or IV	ipilimumab Anti-PD-1 Ipilimumab/nivolumab	60	single factor/multiple factor	5	NLR	OS,PFS
Muhammad Z2	2019	Cohort (retrospective)	Lebanon	120	64 (23–92)	76/44	2011-2017	IIIC or IV	ipilimumab Anti-PD-1 Ipilimumab/nivolumab	60	single factor/multiple factor	2.5	LMR	OS,PFS
Muhammad Z3	2019	Cohort (retrospective)	Lebanon	120	64 (23–92)	76/44	2011-2017	IIIC or IV	ipilimumab Anti-PD-1 Ipilimumab/nivolumab	60	single factor/multiple factor	3	dNLR	OS,PFS
Muhammad Z4	2019	Cohort (retrospective)	Lebanon	120	64 (23–92)	76/44	2011-2017	IIIC or IV	ipilimumab Anti-PD-1 Ipilimumab/nivolumab	60	single factor/multiple factor	5	ANC	OS,PFS
Yuka Matsumura (27)	2022	Cohort (retrospective)	Japan	38	66(42-85)	23/15	2015-2021	NA	Ipilimumab Ipilimumab/nivolumab	20	single factor	3.4	NLR	PFS
Vincent Pozorski (28)	2023	Cohort (retrospective)	USA	183	NA	113/70	2011-2021	III or IV	Ipilimumab/Nivolumab Anti-PD-1 Monotherapy	60	single factor	5	NLR	PFS,OS
P F Ferrucc (29)	2015	Cohort (retrospective)	Italy	69	62(33-87)	42/27	2010-2013	III or IV	Ipilimumab	24	multiple factor	5	NLR	PFS,OS
Franziska K. Krebs (30)	2020	Cohort (retrospective)	Germany	45	70 (27–86)	27/18	2014-2017	IIIC or IV	Ipilimumab Ipilimumab/Nivolumab	24	single factor/multiple factor	289	PLR	OS
Viktoria Anna (31)	2023	Cohort (retrospective)	Germany	138	59 (45.0–72.0)	71/67	2011-2020	III A-D	Anti-PD-1	80	single factor	2	dNLR	OS
Edouard CHASSEUIL (32)	2017	Cohort (prospective)	France	87	71 (27–92)	48/55	2013-2016	IIIC or IV	Nivolumab	NA	multiple factor	NA	NLR	OS,PFS
Jarrett J. Failing (33)	2017	Cohort (retrospective)	USA	133	61 (18–90)	87/46	2012-2015	III or IV	Pembrolizumab	18	single factor/multiple factor	1.7	LMR	OS,PFS
Michael R. Cassidy (34)	2017	Cohort (prospective)	USA	197	NA	125\72	2006-2011	III or IV	Ipilimumab	NA	multiple factor	5	NLR	OS,PFS
P. F. Ferrucci (35)	2016	Cohort (prospective)	Italy	720	61 (17-88)	391/329	2010-2012	NA	ipilimumab	16	single factor/multiple factor	3	dNLR	OS,PFS
P. F. Ferrucci2	2016	Cohort (prospective)	Italy	720	61 (17-88)	391/329	2010-2012	NA	ipilimumab	16	single factor/multiple factor	7.5	ANC	OS,PFS
J. Zaragoza (36)	2015	Cohort (retrospective)	France	58	54.7 (15.6)	33/25	2008-2014	III or IV	ipilimumab	24	multiple factor	4	NLR	OS
C. M. Vila (37)	2021	Cohort (retrospective)	Spain	44	55 (29–76)	24/20	2016-2020	NA	ipilimumab nivolumab	12	multiple factor	5	NLR	OS
C. M. Vila2	2021	Cohort (retrospective)	Spain	44	55 (29–76)	24/20	2016-2020	NA	ipilimumab nivolumab	12	multiple factor	3	dNLR	OS
Paolo A. Asciert (38)	2019	Cohort (retrospective)	USA	71	59 (28–86)	26/45	NA	NA	Nivolumab Pembrolizumab	50	multiple factor	5	NLR	OS,PFS
Mariaelena Capone (18)	2018	Cohort (retrospective)	Italy	97	61 (21–85)	55/42	NA	IV	Nivolumab	24	multiple factor	5	NLR	OS,PFS
Mariaelena Capone2	2018	Cohort (retrospective)	Italy	97	61 (21–85)	55/42	NA	IV	Nivolumab	24	single factor	3	dNLR	OS,PFS
Mariaelena Capone3	2018	Cohort (retrospective)	Italy	97	61 (21–85)	55/42	NA	IV	Nivolumab	24	single factor	5.4	ANC	OS,PFS
Tanja Mesti (39)	2023	Cohort (retrospective)	Slovenia	129	66.2 (30.1–84.5)	84/53	2018-2020	III or IV	Pembrolizumab Nivolumab Nivolumab/Ipilimumab	36	multiple factor	2	NLR	OS,PFS
Tanja Mesti2	2023	Cohort (retrospective)	Slovenia	129	66.2 (30.1–84.5)	84/53	2018-2020	III or IV	Pembrolizumab Nivolumab Nivolumab/Ipilimumab	36	multiple factor	180	PLR	OS,PFS
Michele Guida (40)	2022	Cohort (retrospective)	Italy	272	67.0 (55.0-75.0)	172/100	2011-2019	NA	ipilimumab nivolumab/ipilimumab	40	multiple factor	0.86	NLR	OS,PFS
Michele Guida2	2022	Cohort (retrospective)	Italy	272	67.0 (55.0-75.0)	172/100	2011-2019	NA	ipilimumab nivolumab/ipilimumab	40	multiple factor	22.85	PLR	OS,PFS
Jindrich Kopecky (41)	2022	Cohort (retrospective)	Czech Republic	20	66.5 (35–80)	11/9	2012-2020	NA	nivolumab/pembrolizumab	12	single factor/multiple factor	3	NLR	OS,PFS
Jindrich Kopecky2	2022	Cohort (retrospective)	Czech Republic	20	66.5 (35–80)	11/9	2012-2020	NA	nivolumab/pembrolizumab	12	single factor/multiple factor	160	PLR	OS,PFS
Jindrich Kopecky3	2022	Cohort (retrospective)	Czech Republic	20	66.5 (35–80)	11/9	2012-2020	NA	nivolumab/pembrolizumab	12	single factor/multiple factor	2	LMR	OS,PFS
Umang Swami (42)	2020	Cohort (retrospective)	USA	169	63(24-98)	110/59	2012-2017	NA	Anti-PD-1	60	single factor	4	NLR	OS,PFS
Umang Swami2	2020	Cohort (retrospective)	USA	169	63(24-98)	110/59	2012-2017	NA	Anti-PD-1	60	single factor	1	ANC	OS,PFS
Samuel Rosner (43)	2018	Cohort (retrospective)	USA	209	60.5 (22.0–86.4)	124/85	NA	III or IV	nivolumab/ipilimumab	48	single factor	4.73	NLR	OS
Xue Bai (44)	2021	Cohort (prospective)	China	89	53 (27-78)	44/45	2016-2018	III A-C	Pembrolizumab Camrelizumab	24	multiple factor	1.99	NLR	PFS
Xue Bai2	2021	Cohort (prospective)	China	89	53 (27-78)	44/45	2016-2018	III A-C	Pembrolizumab Camrelizumab	24	multiple factor	0.9	dNLR	PFS
A. Hernando-Calvo1 (45)	2020	Cohort (retrospective)	Spain	52	62.8(15.1-89.3)	31/21	2014-2017	NA	Nivolumab/Pembrolizumab	24	multiple factor	2.5	dNLR	OS
Yoshio Nakamura (46)	2016	Cohort (retrospective)	Japan	98	66.5 (17-93)	52/46	2014-2016	III or IV	nivolumab	24	single factor	4	ANC	PFS

(NLR, Neutrophil-lymphocyteratio; PLR, Platelet-lymphocyteratio; LMR, lymphocyte-monocyteratio; dNLR, Derived neutrophil to lymphocyte ratio; ANC, absolute neutrophil count; OS, overallsurvival; PFS, progression-freesurvival; anti-PD-1, ogrammeddeath-(ligands)1; NA, Not mentioned in the original article)

### The quality assessment of the included studies

The quality of the included cohort studies was evaluated using the Newcastle-OttawaScale(NOS)for quality and the overall quality was rated as good, with the results shown in **Table 2**.

**Table 2:** NOS quality evaluation table.

Study	Selection	Comparability	Outcomes	Total
	1234		123	
Muhammad Z	★★★	★★	★★	7
Yuka Matsumura	★★★	★	★★★	7
Vincent Pozorski	★★★	★	★★★	7
P F Ferrucc	★★★	★★	★★	7
Franziska K. Krebs	★★★	★★	★★★	8
Viktoria Anna	★★★	★	★★★	7
Edouard CHASSEUIL	★★★★	★★	★★★	9
Jarrett J. Failing	★★★	★	★★	6
Michael R. Cassidy	★★★★	★★	★★★	9
P. F. Ferrucci	★★★★	★★	★★★	9
J. Zaragoza	★★★	★★	★★	7
C. M. Vila	★★★	★★	★★★	8
Paolo A. Ascierto	★★★	★★	★★	7
Mariaelena Capone	★★★	★★	★★★	8
Tanja Mesti	★★	★★	★★★	7
Michele Guida	★★★	★★	★★★	8
Jindrich Kopecky	★★★	★★	★★★	8
Umang Swami	★★	★	★★★	6
Samuel Rosner	★★★	★	★★★	7
Xue Bai	★★★★	★★	★★★	9
A. Hernando-Calvo	★★★	★★	★★★	8
Yoshio Nakamura	★★★	★	★★★	7

(★ represents the score, and one ★ is one point)

### Meta-analysis results

#### Overall survival

**Figure 2:**
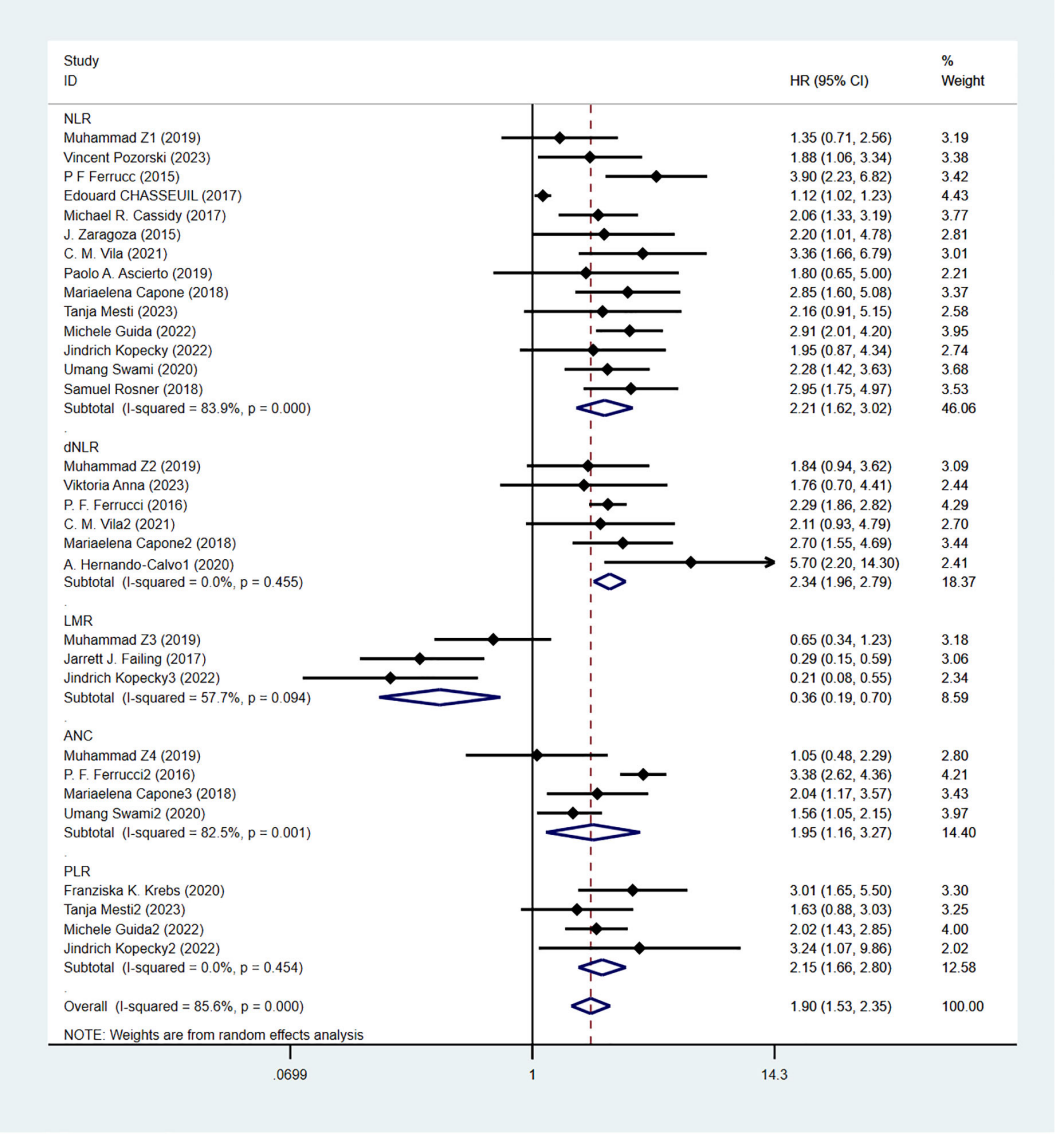
Forest plot of overall survival(OS) data.

Overall survival was reported in 20 studies. **Figure 2** shows the risk-ratio forest plots identified in 20 studies. A random-effects model was used for meta-analysis, taking into account the large heterogeneity between studies (P < 0.001,I²=89.2%). Analysis results showed that high levels of NLR, PLR, dNLR, ANC were associated with poor OS: [HR = 2.21, 95% CI (1.62, 3.02), P < 0.001], [HR = 2.15, 95% CI (1.66, 2.80), P < 0.001], [HR = 2.34, 95% CI (1.96, 2.79), P < 0.001], [HR = 1.95, 95% CI (1.16,3.27),P < 0.001]; In addition, high levels of LMR were associated with OS benefit [HR=0.36, 95%CI (0.19,0.70),P < 0.001]. Considering the existence of large heterogeneity, sensitivity analysis was carried out, and it was found that Umang Swami2 and Edouard CHASSEUIL were the sources of heterogeneity. After excluding the two studies, the heterogeneity of NLR group decreased from 84% to 0%, which may be due to differences in research types and survival analysis methods. The critical value of NLR is not mentioned in the article.

#### Progression-free survival

**Figure 3:**
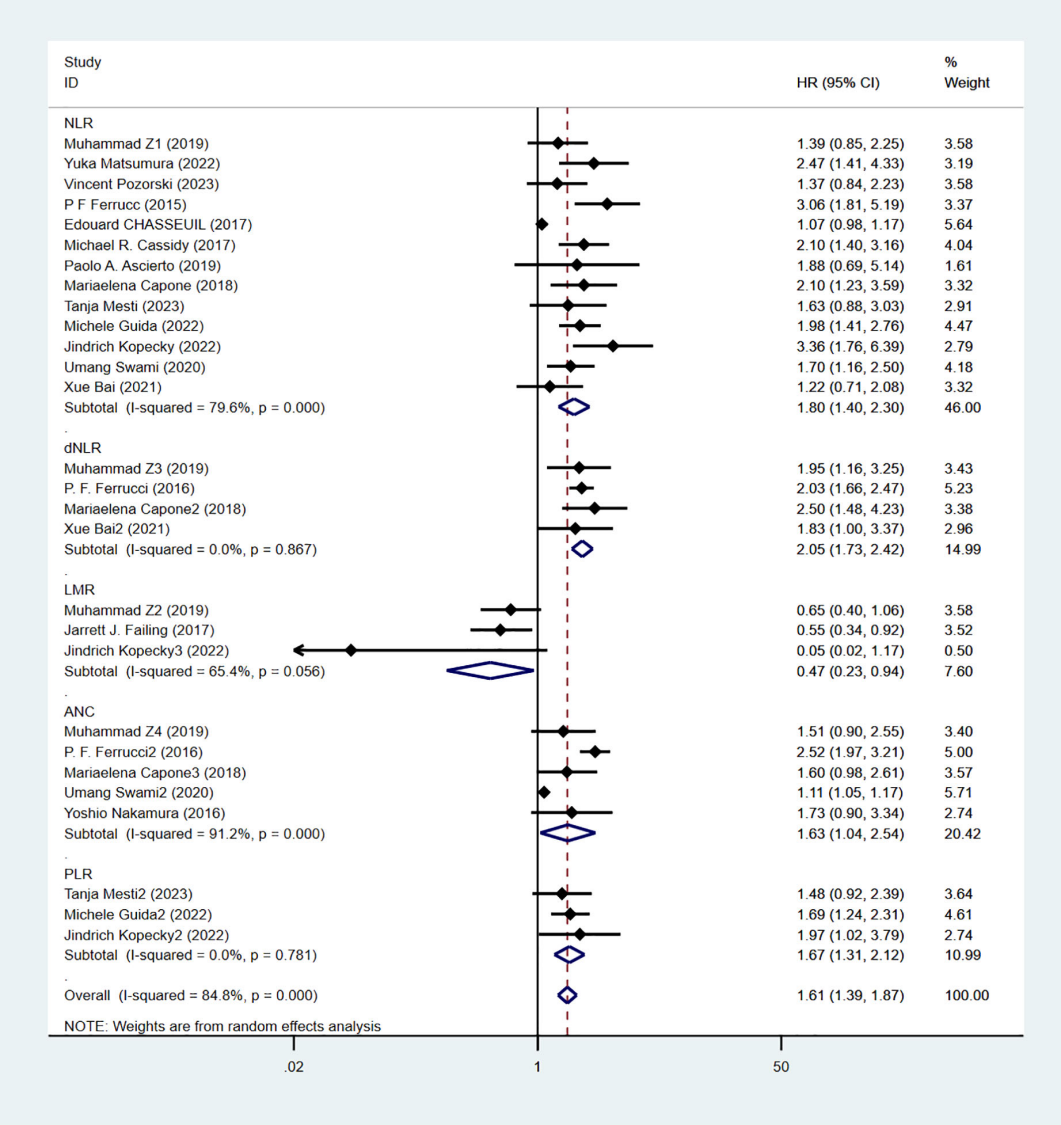
Forest plot of progression-free survival(PFS) data.

Progression-free survival was reported in 16 studies. **Figure 3** shows the risk-ratio forest plots identified in 16 studies. Considering the large heterogeneity between studies (P < 0.01,I²=84.8%), a random effects model was used for meta-analysis. Analysis results showed that high levels of NLR, PLR, dNLR, ANC were all associated with poor PFS: [HR = 1.80, 95% CI (1.40, 2.30), P < 0.001], [HR = 1.67, 95% CI (1.31, 2.12), P < 0.001], [HR = 2.05, 95% CI (1.73, 2.42), P < 0.001], [HR = 1.63, 95% CI (1.04,2.54),P=0.032]; In addition, high levels of LMR were associated with PFS benefit [HR=0.56,95%CI(0.40,0.79),P=0.034]. Sensitivity analysis was also used to explore the sources of heterogeneity, and it was found that Umang Swami2 and Edouard CHASSEUIL were the sources of heterogeneity. After excluding the two studies, the heterogeneity of the NLR group decreased from 79.6% to 21%, and the reasons were analyzed as above.

### Sensitive analysis

**Figure 4:**
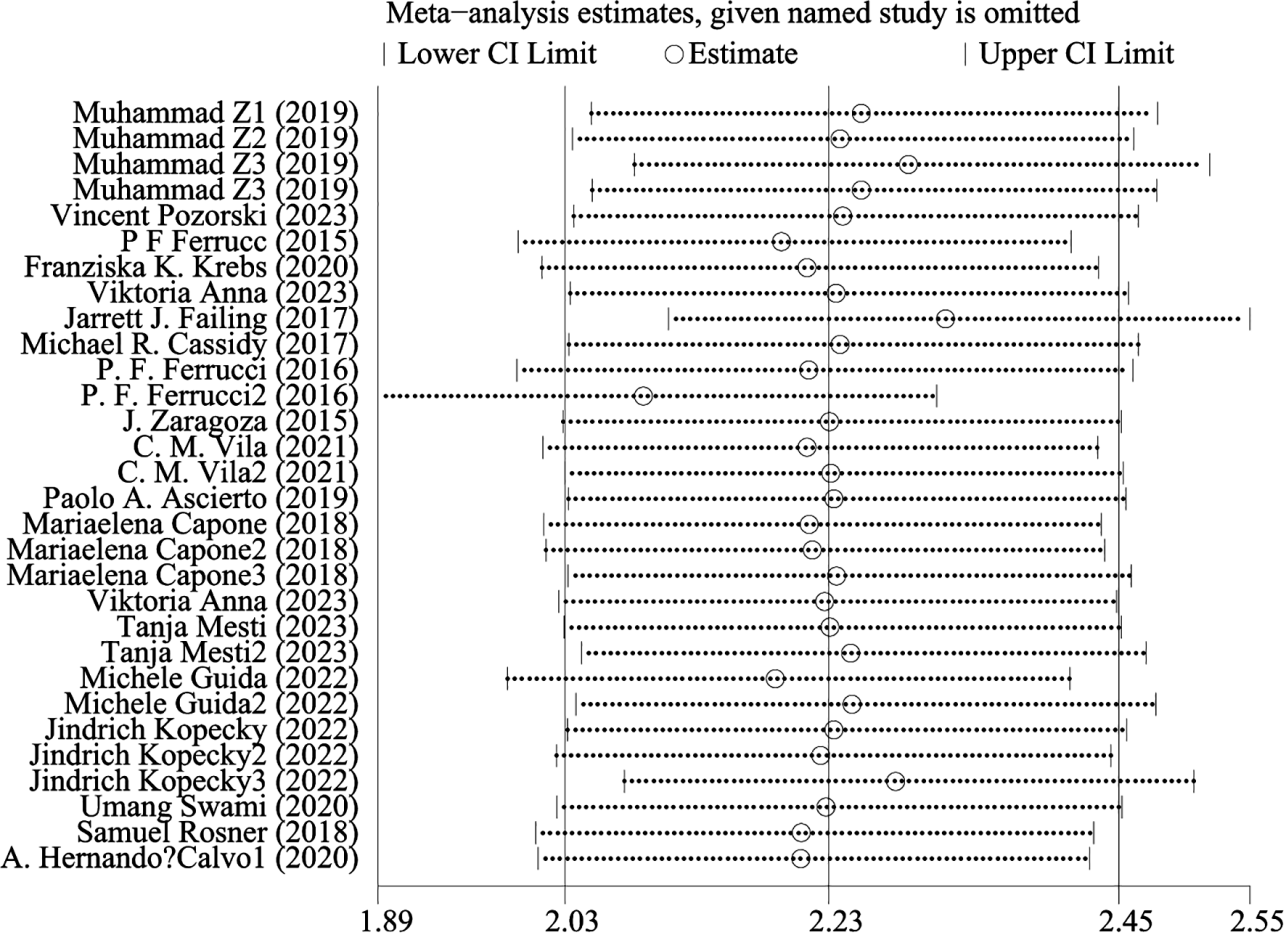
Sensitivity analysis of OS.

**Figure 5:**
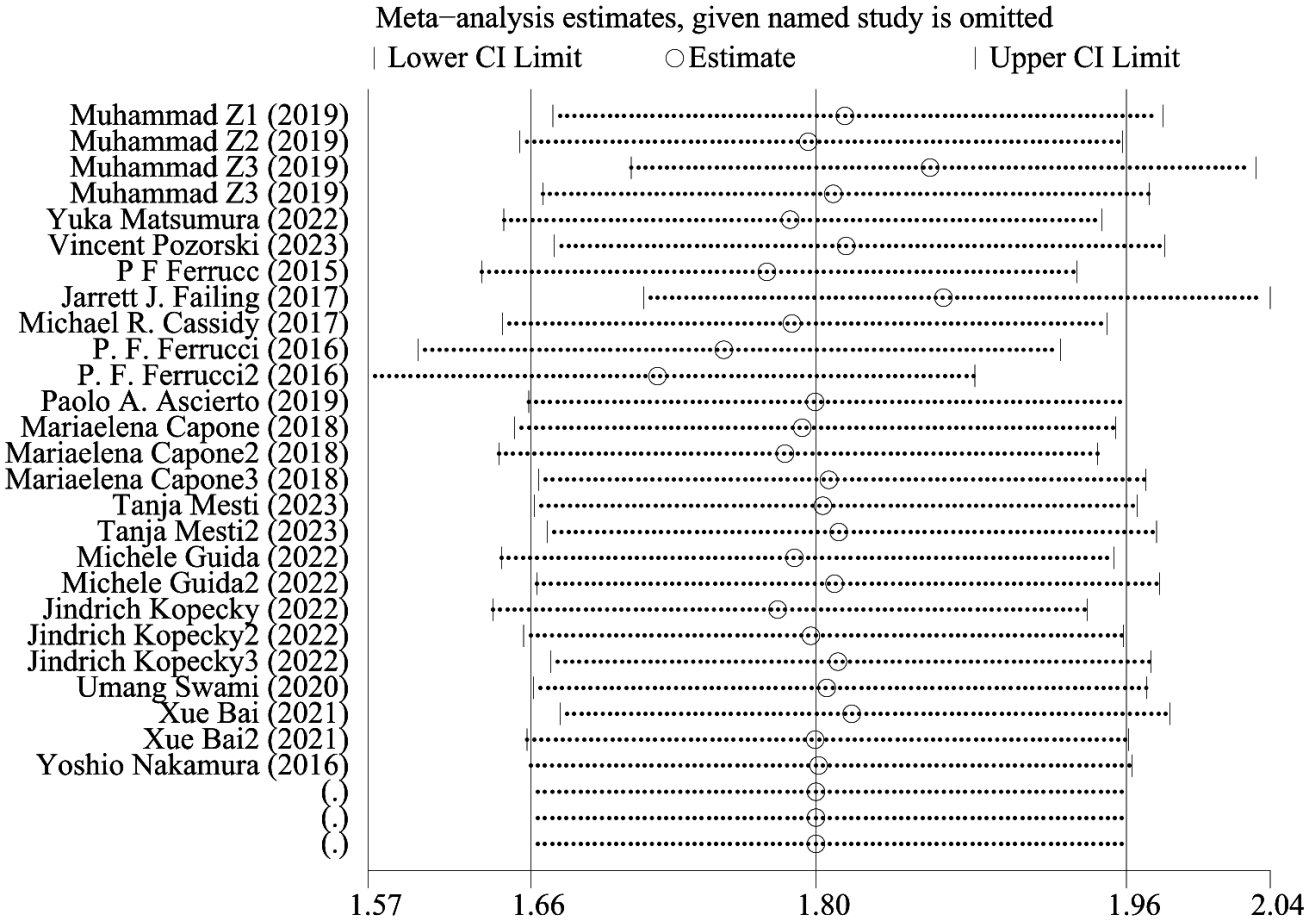
Sensitivity analysis of PFS.

**Figure 4** shows OS sensitivity analysis. After deleting Umang Swami2 and Edouard CHASSEUIL, the model is robust and reliable. **Figure 5** shows the sensitivity analysis of PFS. The sensitivity is low, indicating that the model is robust and reliable.

### Publication bias

**Figure 6:**
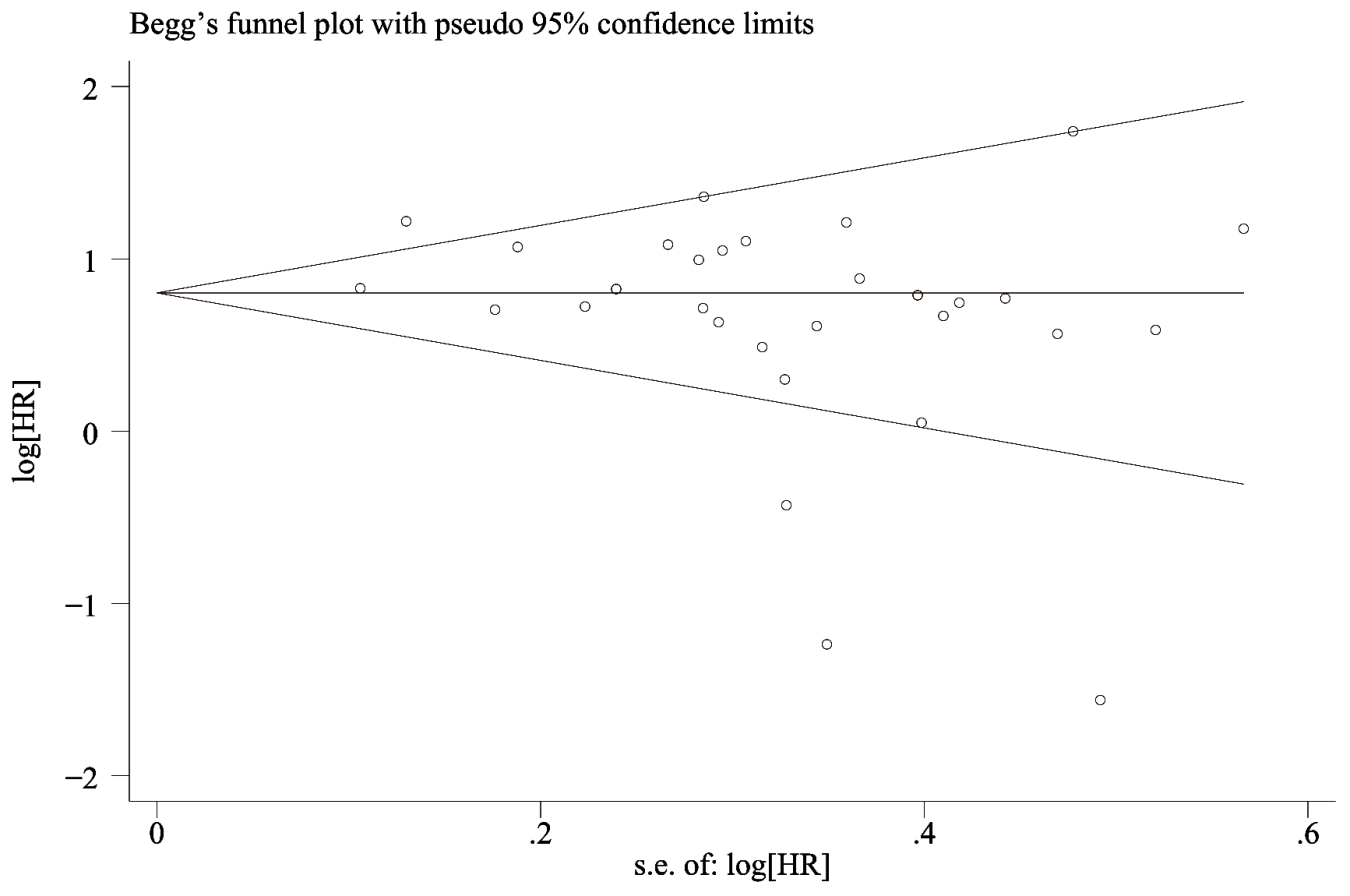
Funnel plot for the evaluation of publication bias for OS.

**Figure 7:**
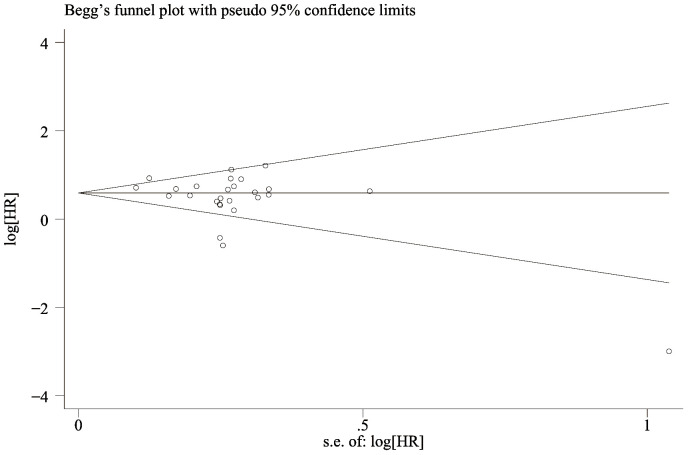
Funnel plot for the evaluation of publication bias for PFS.

The funnel-plot of OS and PFS was evaluated for publication bias, and the results showed that the overall survival rate (**Figure 6**) Egger’s P=0.066 and Begg’s P=0.134, indicating no significant publication bias. There was no significant asymmetry in the shape of the funnel plot, and all studies were within 95%CI range. PFS (**Figure 7**) Egger’s P=0.062 and Begg’s P=0.724 indicate that there is no significant publication bias (P > 0.05). There was no significant asymmetry in the shape of the funnel plot, and all studies were within 95%CI range. ,

### Subgroup analysis

To determine the source of OS and PFS heterogeneity, we conducted subgroup analysis for NLR, dNLR, PLR, and ANC, respectively. Considering that the sample size of the LMR group was only 3 studies, no subgroup analysis was conducted for the LMR group. The results show that high NLR is an important prognostic factor affecting patients’ OS and PFS, and different regions and study types may be the source of heterogeneity, which is similar to the conclusion of our sensitivity analysis. High dNLR and PLR were important prognostic factors for OS and PFS, independent of country, sample size, cut-off value, study type, follow-up duration, and combination drugs. High ANC was also a prognostic factor for OS and PFS. Subgroup analysis showed that sample size, critical value and drug combination were the causes of high heterogeneity, and the results of subgroup analysis were shown in **Table 3**.

**Table 3:** The HR for OS and PFS of NLR,dNLR,PLR,ANC was pooled in subgroup analyses.

NLR								
Subgroup	PFS				OS			
	Study	HR [95%CI]	P value	I^2^	Study	HR [95%CI]	P value	I^2^
Country								
Asia	6	1.93 (1.49,2.49)	P<0.001	40.4	4	2.65 (1.81,3.89)	P<0.001	52.6
Europe	3	1.71 (0.86,3.39)	P=0.127	85.1	5	1.91 (1.14,3.21)	P=0.014	74.1
North America	4	1.74 (1.38,2.21)	P<0.001	0	5	2.23 (1.75,2.83)	P<0.001	0
Sample size								
<100	7	1.95 (1.25,3.03)	P=0.003	84.7	6	2.02 (1.22,3.32)	P=0.006	78.5
≥100	6	1.74 (1.46,2.03)	P<0.001	0	8	2.41 (1.96,2.97)	P<0.001	21.2
cut-off								
≥3	9	2.00 (1.64,2.45)	P<0.001	25.7	11	2.37 (1.98,2.83)	P<0.001	0
<3	3	1.68 (1.27,2.24)	P<0.001	12.1	2	2.78 (1.98,3.90)	P<0.001	0
Study design								
Retrospective	10	1.95 (1.63,2.33)	P<0.001	18.1	11	1.90 (1.61,2.25)	P<0.001	0
Prospective	3	1.37 (0.88,2.13)	P=0.164	80.4	2	2.82 (1.84,4.32)	P<0.001	56.4
Follow-up								
≤24	5	2.27 (1.60,3.24)	P<0.001	50.1	5	2.93 (2.18,3.94)	P<0.001	19.7
>24	6	1.67 (1.38,2.02)	P<0.001	0	7	2.34 (1.90,2.88)	P<0.001	0
Combined medication								
Monotherapy	5	1.83 (1.18,2.84)	P=0.007	87.8	6	2.18 (1.34,3.54)	P=0.002	88.2
Combined therapy	8	1.77 (1.42,2.21)	P<0.001	26.5	8	2.40 (1.94,2.96)	P<0.001	0
**dNLR**								
Subgroup	PFS				OS			
	Study	HR [95%CI]	P value	I^2^	Study	HR [95%CI]	P value	I^2^
Country								
Asia	4	2.05 (1.73,2.42)	P<0.001	0	3	2.29 (1.90,2.77)	P<0.001	0
Europe	0	NA	NA	NA	3	2.72 (1.36,5.44)	P=0.005	44.9
Sample size								
<100	3	2.06 (1.73,2.46)	P<0.001	0	4	2.52 (1.92,3.30)	P=0.009	19.9
≥100	1	1.95 (1.16,3.26)	P=0.011	NA	2	1.81 (1.05,3.12)	P=0.032	0
cut-off								
≥3	3	2.07 (1.74,2.46)	P<0.001	0	4	2.28 (1.90,2.74)	P<0.001	0
<3	1	1.83 (1.00,3.37)	P=0.051	NA	2	3.16 (1.00,9.99)	P=0.05	67.5
Follow-up								
≤24	3	2.07 (1.74,2.46)	P<0.001	0	4	2.52 (1.92,3.30)	P<0.001	19.9
>24	1	1.83 (1.00,3.37)	P=0.051	NA	2	1.81 (1.05,3.12)	P=0.032	0
Combined medication								
Monotherapy	2	2.08 (1.73,2.50)	P<0.001	0	3	2.31 (1.91,2.79)	P<0.001	0
Combined therapy	2	1.90 (1.28,2.81)	P=0.001	0	3	2.65 (1.38,5.07)	P=0.003	49.2
**PLR**								
Subgroup	PFS				OS			
	Study	HR [95%CI]	P value	I^2^	Study	HR [95%CI]	P value	I^2^
Country								
Asia	2	1.66 (1.31,2.12)	P=0.013	NA	1	2.02 (1.43,2.85)	P<0.001	NA
Europe	1	1.63 (1.11,2.40)	P=0.001	13.6	3	2.36 (1.52,3.66)	P<0.001	13.6
Sample size								
<100	2	1.66 (1.31,2.12)	P=0.013	0	2	3.06 (1.80,5.20)	P<0.001	0
≥100	1	1.63 (1.11,2.40)	P=0.001	NA	2	1.92 (1.42,2.60)	P<0.001	0
cut-off								
≥200	0	NA	NA	NA	1	3.01 (1.65,5.50)	P<0.001	1
<200	3	1.67 (1.31,2.12)	P<0.001	0	3	1.99 (1.49,2.66)	P<0.001	3
Follow-up								
≤24	1	1.97 (1.02,3.80)	P=0.043	NA	2	3.06 (1.80,5.20)	P<0.001	2
>24	2	1.62 (1.25,2.11)	P<0.001	0	2	1.92 (1.42,2.60)	P<0.001	2
Combined medication								
Monotherapy	0	NA	NA	NA	0	NA	NA	NA
Combined therapy	3	1.67 (1.31,2.12)	P<0.001	0	4	2.15 (1.66,2.80)	P<0.001	0
**ANC**								
Subgroup	PFS				OS			
	Study	HR [95%CI]	P value	I^2^	Study	HR [95%CI]	P value	I^2^
Country								
Asia	4	1.94 (1.44,2.60)	P<0.001	42.2	3	2.10 (1.11,3.98)	P=0.022	78.7
North America	1	1.11 (1.05,1.17)	P<0.001	NA	1	1.10 (1.05,1.15)	P<0.001	NA
Sample size								
<100	2	1.65 (1.11,2.44)	P=0.013	0	1	2.04 (1.17,3.06)	P=0.012	97.2
≥100	3	1.61 (0.87,2.98)	P=0.129	95.3	3	1.61 (0.67,3.89)	P=0.287	NA
cut-off								
≤5	3	1.30 (0.96,1.75)	P=0.09	47.8	2	1.40 (0.78,3.54)	P=0.261	78.6
>5	2	2.06 (1.26,3.36)	P=0.004	67.2	2	2.00 (0.64,6.26)	P=0.233	87.1
Follow-up								
≤24	3	2.08 (1.51,2.85)	P<0.001	39.5	2	2.80 (1.73,4.52)	P<0.001	61.6
>24	2	1.16 (0.94,1.42)	P=0.164	24.7	2	1.10 (1.05,1.15)	P<0.001	0
Combined medication								
Monotherapy	4	1.66 (0.98,2.79)	P=0.059	93.2	3	1.95 (0.83,4.58)	P=0.125	97.4
Combined therapy	1	1.51 (0.90,2.54)	P=0.121	NA	1	1.05 (0.48,2.29)	P=0.903	NA

NA, not applicable.

### Meta regression

According to the results of the forest map, the NLR group was the main cause of heterogeneity. Therefore, we conducted a meta-regression analysis on the NLR group. **Table 4** shows the results of univariate and multivariate meta-regression, and studies the factors affecting OS. The results of multivariate analysis suggested that study type may be the source of heterogeneity (P < 0.05), which is the same as the results of our sensitivity analysis and subgroup analysis.

**Table 4:** Univariate and multivariate regression.

Covariate	Univariable					Multivariable				
	Coefficients	Lower bound	Upper bound	Std. error	p-Value	Coefficients	Lower bound	Upper bound	Std. error	p-Value
Male	0.0006	-0.003	0.004	0.001	0.724	-0.003	-0.297	0.023	0.013	0.818
Size	0.0004	-0.002	0.002	0.001	0.701	0.003	-0.014	-0.020	0.008	0.729
Cut	-0.033	-1.160	0.094	0.058	0.583	0.006	-0.178	0.192	0.094	0.942
Research	-1.104	-0.804	0.596	0.321	0.751	-0.559	-1.046	-0.072	0.248	0.024

## Discussion

Melanoma is a malignant tumor that originates from primitive nerve cells and results from the over proliferation of abnormal melanocytes. While it primarily occurs in the skin, melanoma can also be found in other locations, including the eyes, ears, meninges, gastrointestinal tract, oral cavity, genitals, and the mucous membranes of the sinuses (47), In most populations, cutaneous melanomas represent over 90% of melanoma diagnoses. Malignant melanoma can develop from benign cutaneous melanomas and may be triggered by various factors, including ultraviolet radiation and genetic predisposition. Ultraviolet radiation, known to induce DNA mutations, is regarded as the primary environmental factor contributing to melanoma development (48). Its clinical characteristics are highly invasive, highly malignant, frequently recurring and easily metastasized, and its incidence is on the rise worldwide (49). Currently, the clinical diagnosis and treatment of melanoma are conducted by a multidisciplinary team (MDT) within the framework of integrative oncology. The primary treatment strategy involves extensive surgical resection, while immunotherapy, chemotherapy, and radiotherapy serve as the principal modalities for patients with deeply metastasized tumors or those whose cancer has spread to the lymph nodes (50). Due to melanoma’s high susceptibility to metastasis, most patients are diagnosed at intermediate to advanced stages, where surgical treatment becomes less effective and sensitivity to radiotherapy is low. This results in poor efficacy and the development of drug resistance. The objective remission rate (ORR) for first-line chemotherapy regimens based on the alkylating agents dacarbazine and temozolomide is less than 20%, with a 5-year overall survival (OS) rate of under 5%. Other chemotherapeutic agents have not demonstrated improved long-term survival, underscoring the urgent need for novel therapeutic approaches (51). The hallmarks of melanoma include mutations in genes associated with the mitogen-activated protein kinase (MAPK) pathway or the over-activation of proteins, which result in increased tumor cell proliferation and invasive capabilities, as well as immunosuppression within the tumor microenvironment (TME). The immune effects in the TME are primarily mediated by adaptive immune cells, which undergo a series of proliferation and differentiation processes in response to antigen stimulation. The effector T cells produced are capable of specifically binding to target cells within the organism, leading to their cleavage and subsequent death, thereby achieving the antitumor effect (52) (53).The latest ECSO guidelines recommend immune checkpoint blockade, either with anti-PD-1 alone or in combination with anti-CTLA-4, as a first-line treatment for patients with unresectable stage III and IV melanoma. Immune checkpoint inhibitors (ICIs) enhance the host’s immune response to tumors by inhibiting negative immunomodulatory molecules on T cells, thereby activating the immune system. This therapeutic approach has significantly improved clinical outcomes (54). Immunotherapy has significantly improved survival rates for melanoma patients, with a 5-year overall survival rate of up to 93.5%. Specifically, the overall survival rates are 73.9% for stage III and 35.1% for stage IV melanoma patients (55). The results of several Meta-analyses have also demonstrated better survival rates with ICIs in the treatment of progressive melanoma (56) (57). CTLA-4, an inhibitory receptor expressed by T cells, is a cellular antigen primarily derived from human cells. The T cell receptor recruits phosphatases that inhibit the activation of transcription factors and ubiquitin ligases associated with T cell activity, thereby attenuating signaling (58).Additionally, CTLA-4, another immune transmembrane receptor found on T lymphocytes, is upregulated during T cell activation and provides negative regulation of the immune system. It competes with CD28 for binding to B7 ligands. When CTLA-4 binds to B7 instead of CD28, it results in a loss of immune-reactive enzyme activity in T cells (59). CTLA-4 inhibitors target the binding of CTLA-4 to its ligands, CD80 and CD86, thereby blocking the interaction between CTLA-4 and these ligands. This blockade enhances antitumor T-cell activity. Approved drugs in this category include tremelimumab and ipilimumab (60). In the development of melanoma, tumor cells induce T-cell catabolism by binding to PD-1, a process that is activated through the phosphorylation of PD-1 and its ligand PD-L1 by the protein tyrosine kinase Lck. This interaction subsequently recruits the tyrosine phosphatase Shp2, which mediates the dephosphorylation of the T-cell receptor (TCR) and CD28, thereby inhibiting T-cell-associated signaling. Consequently, tumor cells proliferate by evading T-cell-mediated killing, contributing to the progression of melanoma (61) (62).PD-1/PD-L1 inhibitors bind to PD-1 or PD-L1, respectively, blocking the interaction between these two proteins. This action restores the recognition and cytotoxic effects of immune cells, thereby reducing the incidence of immune escape by tumor cells. Consequently, T cells are induced to exert their killing effects, leading to the elimination of tumor cells. Approved drugs in this category include nivolumab and pembrolizumab. In a study by Larkin J et al. (63) comparing the efficacy of nivolumab and dacarbazine in the treatment of unresectable stage III or IV melanoma, nivolumab treatment showed a significant clinical benefit, with a higher ORR and a higher median OS in the nivolumab group compared to the chemotherapy group (31.7% vs. 10.6%, respectively, and 16 months vs. 14 months).

Although immune checkpoint inhibitors, such as anti-CTLA-4 and PD-1, have demonstrated promising anti-tumor effects in the clinical treatment of melanoma, a subset of patients does not respond to these immunotherapeutic agents. Clinical trials indicate that the overall response rate to PD-1 antibody treatment for solid tumors is approximately 20%, while the response rate for combinations of multiple immune checkpoint inhibitors has not surpassed 50% (64, 65). It was found that tumor BRAF, NRAS, HRAS gene mutation status, Ki67, P16, PTEN protein expression levels, miRNA, lncRNA non-coding RNA mutation sites are potential prognostic predictors of malignant melanoma (66, 67), However, clinical testing for the aforementioned markers is expensive and controversial regarding their prognostic accuracy, rendering them unsuitable for routine screening of melanoma patients. Therefore, identifying cost-effective and easily accessible biomarkers is particularly important. Analyzing these biomarkers in combination can assist in identifying patient groups that will benefit most from immunotherapy and optimizing drug regimens. Inflammatory-related factors, such as cytokines, inflammatory cells, and chemokines, are present in the microenvironment of all early-stage tumors, and the persistent inflammatory response may significantly drive tumorigenesis (68). The overexpression of inflammatory mediators promotes angiogenesis, induces cellular mutations and DNA damage, triggers inflammatory cascades, and results in tissue atrophy. The inflammatory state of the body leads to an impaired immune response, which contributes, either directly or indirectly, to tumorigenesis, invasion, and metastasis (69, 70). Therefore, predictive biomarkers that reflect the inflammatory response, such as neutrophil, lymphocyte, and platelet counts, may be useful in clinical decision-making for managing melanoma patients. These markers are economically accessible and noninvasive. Although many studies have examined the prognostic significance of inflammatory markers in melanoma patients treated with ICIs, the results remain controversial. Therefore, we conducted this meta-analysis, which showed that levels of NLR, PLR, dNLR, and ANC were all independent predictors of OS and PFS in melanoma patients treated with ICIs: High level of NLR was associated with poor OS and PFS, and the combined HR was OS[HR=2.21,95%CI(1.62,3.02), P < 0.001] and PFS[HR=1.80,95%CI(1.40,2.30), P < 0.001], respectively. High levels of PLR were associated with poor OS and PFS, and the combined HR was OS[HR=2.15,95%CI(1.66,2.80), P < 0.001] and PFS[HR=1.67,95%CI(1.31,2.12), P < 0.001]. High levels of dNLR were associated with poor OS and PFS, with combined HR being OS[HR=2.34,95%CI(1.96,2.79), P < 0.001] and PFS[HR=2.05,95%CI(1.73,2.42), P < 0.001], respectively. High ANC was associated with poor OS and PFS, and combined HR was OS[HR=1.95,95%CI(1.16,3.27), P < 0.001] and PFS[HR=1.63,95%CI(1.04,2.54),P=0.032], respectively. Increased LMR was associated with prolonged OS and PFS, with combined HR being OS[HR=0.36, 95%CI(0.19,0.70), P < 0.001] and PFS[HR=0.56,95%CI(0.40,0.79), P=0.034], respectively. Meanwhile, the analysis showed that high dNLR resulted in worse OS and PFS. Subgroup analyses showed that region, threshold, and whether monotherapy or combination were factors contributing to differences and bias, and that differences in the thresholds of inflammatory markers may cause differences in the sensitivity of prognostic prediction. The results of a study by Ari VanderWalde et al. (71) validated our analysis: nabulizumab in combination with ibritumomab significantly improved progression-free survival compared with ibritumomab alone, with objective remission rates of 28% and 9%, respectively. The NLR serves a dual role in both the promotion and suppression of cancer within the tumor microenvironment. Neutrophils can be classified into two distinct phenotypes based on their function: high-density neutrophils (HDNs) and low-density neutrophils (LDNs) (72). The HDN phenotype enhances antitumor effects by directly targeting tumor cells and stimulating T-cell-mediated immunity. Conversely, the LDN phenotype suppresses antitumor T-cell responses through various mechanisms: it releases arginases that degrade antitumor factors, produces leukotrienes that promote metastasis-initiating cells, and induces angiogenesis via the release of vascular endothelial growth factor (VEGF). These actions undermine the immune system, allowing tumor cells to evade detection and thereby facilitating tumor progression and metastasis (73, 74). Tumorigenesis is characterized as a chronic inflammatory process. During the initial stages of inflammation, neutrophils predominantly display the HDN phenotype, whereas the LDN phenotype becomes more prevalent as inflammation subsides. As a result, an elevated ANC serves as a poor prognostic indicator. Lymphocytes, which are responsible for mediating both cellular and humoral immune responses, play a pivotal role in defending against pathogens, eliminating tumor cells, and regulating immune responses through the induction of apoptosis and the inhibition of tumor cell proliferation and migration (75).Elevated levels of the NLR and the dNLR indicate a poor prognosis. The PLR reflects the role of platelets, which are produced by megakaryocytes in the bone marrow. Platelets are among the first cells to aggregate at the site of injury and play a significant role in tumor metastasis as ‘first responders. Their hemostatic function is compromised and exploited by tumor cells within the tumor microenvironment. Platelets adhere to tumor cells to form aggregates, which protect these cells from high-flow shear stress and immune attacks, thereby promoting tumor progression, invasion, and metastasis (76, 77). An elevated PLR indicates a poorer prognosis. The LMR assesses the balance between lymphocytes and monocytes. Monocytes release various pro-inflammatory cytokines, including interleukins IL-1, IL-6, IL-10, and TNF-α, which are linked to reduced survival and a worse prognosis in patients with malignant tumors (78). An elevated PLR is associated with a poorer prognosis. The LMR evaluates the balance between lymphocytes and monocytes. Monocytes secrete a variety of pro-inflammatory cytokines, such as interleukins IL-1, IL-6, IL-10, and TNF-α, which have been correlated with reduced survival and a worse prognosis in patients with malignant tumors (79). Therefore, the higher LMR its level, suggests that the better the immune status of the body, the better the ability to monitor tumor cells, the better the prognosis. Increasing research evidence suggests that ICIs play a crucial role in the treatment of advanced melanoma, demonstrating irreplaceable functions in various contexts, whether as monotherapy or in combination therapy. The development of safe, effective, economical, and highly specific immunotherapy circulating biomarkers can not only dynamically monitor the therapeutic effects of ICIs in patients but also provide timely feedback on the efficacy of immune drugs. Furthermore, these biomarkers can flexibly predict clinical outcomes for patients undergoing immunotherapy and facilitate the early identification of benefit groups, thereby enhancing the clinical application value of ICIs.

This Meta-analysis of ours has some significant strengths: (1) it is the first assessment of the prognostic predictive ability of peripheral blood inflammatory markers NLR, dNLR, PLR, LMR, and ANC in melanoma patients treated with ICIs, and the search of the literature is more comprehensive; and (2) the data were analyzed in subgroups meticulously, and the findings were fully discussed. However, it is undeniable that there are some shortcomings in our study:(1) there is some heterogeneity among studies, which may be caused by the fact that some studies did not specify the cutoff value;(2)Most of the included studies were retrospective cohort studies, which may introduce confounding bias. Furthermore, the levels of inflammatory markers can be influenced by other conditions, such as infections, and these confounding factors may impact the results. Therefore, we anticipate the emergence of more large-scale, well-designed prospective studies in the future to validate our findings. It is recommended that original studies employ more rigorous designs and methodologies to minimize bias and error. Additionally, researchers should be encouraged to publish their study data and methods for verification and reanalysis by others. Finally, establishing a uniform standard for cutoff values of inflammatory markers is essential to mitigate the risk of bias.

## Conclusion

In melanoma patients receiving immune checkpoint inhibitors (ICIs), the neutrophil-to-lymphocyte ratio (NLR), platelet-to-lymphocyte ratio (PLR), derived neutrophil-to-lymphocyte ratio (dNLR), and absolute neutrophil count (ANC) may serve as effective biomarkers for prognostic prediction. These metrics offer valuable insights for therapeutic decision-making in the context of melanoma immunotherapy. However, further high-quality prospective studies are necessary to validate these findings.

## Data Availability

The original contributions presented in the study are included in the article/supplementary materials, further inquiries can be directed to the corresponding author.
